# Creativity is associated with a characteristic U-shaped function of alpha power changes accompanied by an early increase in functional coupling

**DOI:** 10.3758/s13415-019-00699-y

**Published:** 2019-02-12

**Authors:** Christian Rominger, Ilona Papousek, Corinna M. Perchtold, Mathias Benedek, Elisabeth M. Weiss, Andreas Schwerdtfeger, Andreas Fink

**Affiliations:** Department of Psychology, University of Graz, Univ.-Platz 2, A-8010 Graz, Austria

**Keywords:** Creative ideation, EEG, Upper alpha power, Time-course, Phase locking value

## Abstract

Although there exists increasing knowledge about brain correlates underlying creative ideation in general, the specific neurocognitive mechanisms implicated in different stages of the creative thinking process are still under-researched. Some recent EEG studies suggested that alpha power during creative ideation varies as a function of time, with the highest levels of alpha power after stimulus onset and at the end of the creative thinking process. The main aim of the present study was to replicate and extend this finding by applying an individual differences approach, and by investigating functional coupling between long distance cortical sites during the process of creative ideation. Eighty-six participants performed the Alternate Uses (AU) task during EEG assessment. Results revealed that more original people showed increased alpha power after stimulus onset and before finalizing the process of idea generation. This U-shaped alpha power pattern was accompanied by an early increase in functional communication between frontal and parietal-occipital sites during the creative thinking process, putatively indicating activation of top-down executive control processes. Participants with lower originality showed no significant time-related variation in alpha power and a delayed increase in long distance functional communication. These findings are in line with dual process models of creative ideation and support the idea that increased alpha power at the beginning of the creative ideation process may indicate more associative modes of thinking and memory processes, while the alpha increases at later stages may indicate executive control processes, associated with idea elaboration/evaluation.

## Introduction

Generating original, useful, and innovative ideas is one of the most important and fascinating cognitive skills, relevant in many different contexts and situations in daily life (see, e.g., Beaty, 2015; Boccia, Piccardi, Palermo, Nori, & Palmiero, 2015; Fink et al., 2017; Fink, Bay, et al., 2018; Lopata, Nowicki, & Joanisse, 2017; Papousek, Weiss, et al., 2017; Pinho, Ullen, Castelo-Branco, Fransson, & de Manzano, 2016). As a result of this growing interest, there are an increasing number of cognitive neuroscience studies attempting to unveil potential brain mechanisms associated with creative ideation (see, e.g., Arden, Chavez, Grazioplene, & Jung, 2010; Fink & Benedek, 2014; Jung & Vartanian, 2018; Pidgeon et al., 2016). Though research in this field is still at an early stage, considerable progress has been achieved in unveiling relevant brain mechanisms of divergent thinking by means of event- and task-related (de)synchronization of power in the EEG alpha band (TRP; e.g., Benedek, Bergner, Könen, Fink, & Neubauer, 2011; Benedek, Schickel, Jauk, Fink, & Neubauer, 2014; Fink, Benedek, Grabner, Staudt, & Neubauer, 2007; Fink, Graif, & Neubauer, 2009; Fink, Rominger et al., 2018; Jausovec, 2000; Rominger, Papousek, Perchtold et al., 2018; for an overview see Fink & Benedek, 2014).

Specifically, there is consistent evidence of increased alpha power at bilateral frontal and (right) posterior cortical sites during various divergent thinking demands (Fink & Benedek, 2014). These task-related alpha power increases, somewhat more pronounced in the upper alpha band (~10–12 Hz), are thought to indicate active inhibition of (task-irrelevant) sensory stimuli, internal attention, controlled memory retrieval processes, and the shielding of working memory processes from task-irrelevant information (for overviews see, e.g., Benedek, 2018a; Fink & Benedek, 2014; Fink, Perchtold, & Rominger, 2018). Critically, these neurocognitive functions (i.e., memory retrieval, inhibition, internal attention) might not be equally involved at any time point during the generation of novel ideas. Rather, considering existing behavioral research, it seems reasonable to assume that they are implicated in different stages of the creative ideation process (e.g., generative and exploratory processes, Finke, Ward, & Smith, 1996; see also Beaty, Benedek, Kaufman, & Silvia, 2015; Benedek & Jauk, 2018; Ellamil, Dobson, Beeman, & Christoff, 2012; Fink, Rominger, et al., 2018; Kleinmintz et al., 2018; Pringle & Sowden, 2017; Rominger, Papousek, Perchtold, et al., 2018; Sowden, Pringle, & Gabora, 2015). For instance, Gilhooly, Fioratou, Anthony, and Wynn (2007) found by means of an overt verbal Alternate Uses (AU; Guilford, 1967) task that initial ideas were more often based on memory retrieval, while later ideas were typically based on more complex processes such as imagination and inhibition (see also Cheng, Hu, Jia, & Runco, 2016; Silvia, Nusbaum, & Beaty, 2017). This is congruent with the assumption that at later stages of the creative thinking process, prepotent, obvious, and common ideas are inhibited and memory content is integrated in the generation of new ideas, which presumably leads to more creative outcomes (Beaty & Silvia, 2012; Benedek, Jauk, et al., 2014; Benedek et al., 2018; Cheng et al., 2016; Rominger, Papousek, Weiss, et al., 2018; Wang, Hao, Ku, Grabner, & Fink, 2017; Zabelina, Robinson, Council, & Bresin, 2012).

Despite these behavioral findings, little is known about the time-course of functional patterns of brain activity during creative ideation (but see, e.g., Beaty et al., 2015). EEG techniques are especially suited to investigate such time-related neurocognitive processes during the generation of novel ideas, because of excellent time resolution (Fink et al., 2007). In a first EEG study in this context, Schwab, Benedek, Papousek, Weiss, and Fink (2014) found that the process of generating original alternative uses of objects was reflected in a characteristic time-course of TRP changes in the upper alpha band. Specifically, this study revealed that the generation of more versus fewer original ideas was accompanied by a U-shaped function of alpha power during creative ideation, with higher levels of alpha power at the beginning of idea generation, followed by a decrease and finally by a re-increase in alpha power prior to finalizing the idea (prominently in the right hemisphere). Two more recent studies reported a similar pattern during figural divergent thinking tasks. Jaarsveld et al. (2015) found increased upper alpha TRP changes at the initial and final stage during the process of solving an ill-defined and open problem (i.e., creating a figural intelligence test). In the study by Rominger, Papousek, and Perchtold et al. (2018), the stage of idea elaboration (during a picture completion task) was associated with increased upper alpha TRP changes (compared to the preceding stage of idea generation). In line with relevant behavioral and neuroimaging studies (e.g., Benedek, Schickel, et al., 2014; Gilhooly et al., 2007), the characteristic U-shaped time-course has been interpreted as an initial recall of ideas from memory, followed by an increase in inhibitory control and evaluation/elaboration processes (Jaarsveld et al., 2015; Rominger, Papousek, Perchtold, et al., 2018; Schwab et al., 2014).

While available EEG studies on time- or process-related changes in alpha power during creative ideation used within-subjects research designs (comparing brain activity in response to more vs. fewer original ideas, or conditions that place different demands on creativity), it is still unknown whether interindividual variations in originality are linked to corresponding time-related changes in alpha power as well. Hence, the first aim of the present study was to test whether participants who generate more original ideas in the AU task likewise show the characteristic U-shaped time-related changes of alpha power during creative ideation, as shown in EEG studies employing a within-subject design (e.g., Schwab et al., 2014). As an important extension of available literature, this study also investigated functional coupling between long distance cortical sites during the process of creative ideation. This could facilitate a more comprehensive assessment of the specific kind of neurocognitive mechanisms (e.g., executive control mechanisms) that are implicated in different stages of the creative ideation process. For this purpose, measures of task-related alpha power changes were combined with task-related long distance functional connectivity measures (i.e., phase-locking values; Lachaux, Rodriguez, Martinerie, & Varela, 1999; Varela, Lachaux, Rodriguez, & Martinerie, 2001) between frontal and parietal-occipital areas, which provide an index of neuronal integration and communication within the executive control network. This functional connectivity approach appears to be particularly appropriate in this context since former EEG studies reported long distance communication between brain areas (i.e., frontal to parietal areas) during different creative ideation tasks (Jausovec & Jausovec, 2000; Petsche, 1996; Petsche, Kaplan, von Stein, & Filz, 1997). A more recent fMRI study even indicated, by means of a region-of-interest (ROI)-to-ROI temporal connectivity analysis, a time-course of functional coupling between frontal and parietal areas during the AU task (Beaty et al., 2015), which increases during the final stages of the creative ideation process. Functional coupling between frontal and parietal areas may reflect the involvement of executive control networks in creative ideation (Beaty, Benedek, Silvia, & Schacter, 2016; Ellamil et al., 2012; Gonen-Yaacovi et al., 2013). Hence, in conjointly looking at alpha power and functional connectivity patterns during different stages of the creative thinking process (by dividing the continuous thinking process during an AU-task into three isochronous time intervals), this study targeted a more in-depth understanding of the manifold neurocognitive processes implicated in the generation of creative ideas.

## Methods

### Participants

The total sample consisted of 102 participants. Sixteen participants (15.68%) were excluded from the analyses (see below for detailed information on the exclusion criteria). The final sample consisted of 86 participants (30 men) with an average age of 23.36 years (*SD* = 3.55; min = 18, max = 33). People with major psychiatric disorders/history of major psychiatric disorders according to the Structured Clinical Interview for DSM-IV Axis I Disorders (SCID screening) and people who reported having a neurological disease or using psychoactive medication were not included in the study. All participants were right-handed (assessed by a standardized hand skill test; Steingrüber, 2010), and were requested to refrain from alcohol intake for 12 h and from drinking coffee and other stimulating beverages for 2 h prior to their lab appointment, and to come to the session well rested.

Written informed consent was obtained from all participants. The study was approved by the authorized ethics committee.

### Creative thinking task

The AU task (Guilford, 1967) is a verbal creativity/divergent thinking test used in numerous neuroscientific studies of creativity (e.g., Benedek, Jauk, et al., 2014; Fink et al., 2007; Silvia, Beaty, Nusbaum, Eddington, & Kwapil, 2014). In this task participants are requested to generate as original as possible uses to everyday objects within a given period of time. For the purpose of the present investigation, one self-paced AU task was used where the participants had to produce a single answer (best idea). An instruction to select the best and most appropriate idea was applied in order to increase elaboration and evaluation processes during creative ideation (Goldschmidt, 2016; Runco & Acar, 2012). Therefore, the applied approach strongly focuses on the originality aspect of creativity (Perchtold et al., 2018). The self-paced procedure might more appropriately capture the spontaneous nature of the creative thinking process and allows a more accurate estimation of different stages of the creative ideation process (Benedek, Jauk, et al., 2014; Benedek et al., 2018; Fink, Rominger, et al., 2018; Jauk, Benedek, & Neubauer, 2012).

**Fig. 1:**
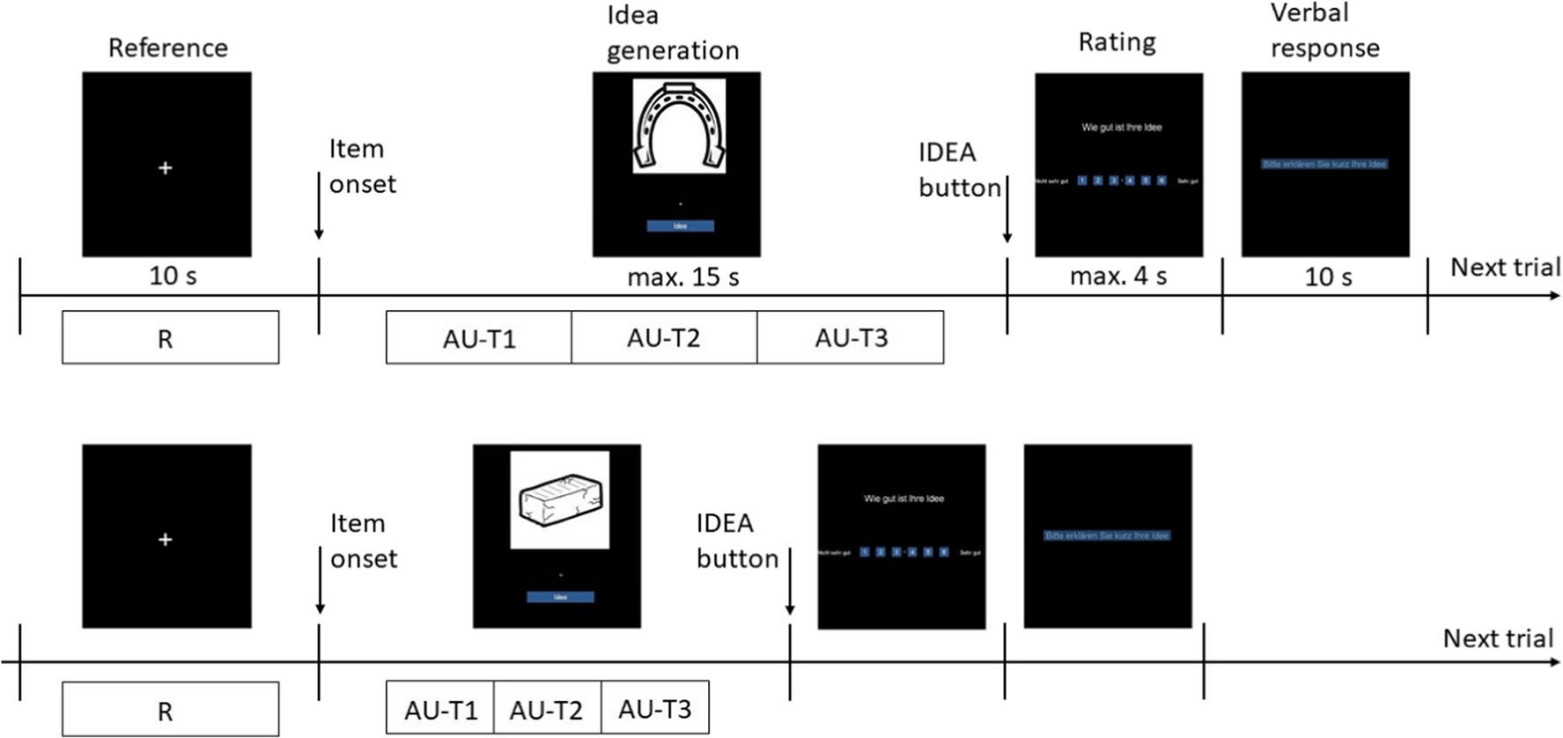
Schematic display of the computerized AU task. An 8-s time period out of the 10-s fixation cross period (“Reference”) served as the reference interval (leaving out the first and the last second). The time period starting 500 ms after item onset until 500 ms before the IDEA button was pressed (“Idea generation”) served as the activation interval, which was further divided into three isochronous sections (AU-T1, AU-T2, AU-T3). The participants rated the subjective originality of their idea from one to six (not original – very original; maximum 4 s) and after their verbal response (10 s, audio recording) the next trial started

The AU task started with a white cross (10 s; reference period) followed by a picture of a common every day object (e.g., umbrella, brick, or key). The picture onset indicated the beginning of the creative ideation period, upon which participants had to generate a single use of this particular object that is as original as possible (e.g., an umbrella as a fruit basket, a key to decorate the Christmas tree, a brick as a pencil holder). The participants had to press the IDEA button as soon as they decided to name their best idea (maximum time until the IDEA button had to be pressed was 15 s, *M* = 6.72 s, *SD* = 2.10 s). After the button-press and before verbalizing their best idea, participants rated this idea on a six-point Likert scale (maximum response time was 4 s; from 1 “not original” to 6 “very original”). This phase was included in order to increase or at least maintain the participants’ effort to produce high-quality ideas for every single item. At the end of each trial, participants described their best idea as quickly as possible (10 s), which was recorded online and later transcribed for analysis. After that a new trial started with a white cross (see Fig. 1 for an illustrative summary of the procedure). The 16 AU items were presented in randomized order.

### Quantification of creative performance

The originality of the produced ideas was rated by three independent and experienced judges (all master students) who were thoroughly instructed on how to perform the ratings. Originality depended on the uniqueness of ideas and if these ideas were possible in principle (no fiction or fantasy). Originality was rated on a four-point Likert scale ranging from “not original” (1) to “very original” (4). This procedure is a common approach in creativity research (cf. Consensual Assessment Technique; Amabile, 1982; see also, e.g., Rominger, Fink, Weiss, Bosch, & Papousek, 2017). The originality ratings showed acceptable inter-rater reliability (ICC (2, k) = .66). All ratings were averaged across raters and items, resulting in one originality measure per participant.

### EEG recordings and analysis

The EEG was recorded by 19 electrodes (Fp1, Fp2, F3, F4, F7, F8, C3, C4, T7, T8, P3, P4, P7, P8, O1, O2, Fz, Cz, Pz), positioned according to the 10–20 system (Brainvision BrainAmp Research Amplifier, Brain Products^TM^; 500-Hz sampling rate) in a separate and quiet room. All participants were instructed not to close their eyes during the AU-task. Behavior compliance was monitored by the use of a video camera. The ground electrode was located on the forehead, the reference electrode on the nose. Vertical and horizontal electro-oculograms were measured with two bipolar channels for horizontal and vertical eye movements. Electrode impedances were kept below 5 kΩ for all electrodes. The data were preprocessed by removing drifts and low-pass filtering (50 Hz).

The g.BSanalyze software (g.tec™, Graz, Austria) was used to manually check the resulting signal for artifacts and to calculate the band power values (μV^2^) by squaring the filtered EEG signals (10–12 Hz; FFT-filter with a window size of 100 samples and an overlap of 99 samples). For estimating the phase-locking values, the convolution between two signals was computed by means of a wavelet centered at a frequency of 11 Hz and a bandwidth of 2 Hz (10–12 Hz). The phase of the convolution was extracted and was used as an index of phase-locking, which varies between 0 (independent signals, no functional coupling) and 1 (constant phase lag between two signals, perfect functional coupling; see, e.g., Lachaux et al., 1999). The phase-locking value separates the effect of the phase component from the amplitude component for a specific frequency band and represents a measure of neuronal integration and functional coupling (Lachaux et al., 1999; Varela et al., 2001).

Only band power and phase-locking values from artifact-free time-intervals were averaged by means of the median. For the TRP and the task-related phase-locking (TRPL) analyses, the 8-s interval from 1 s after onset of the fixation cross until 1 s before its offset served as the reference interval and the period starting 500 ms after stimulus onset until 500 ms before the IDEA button was pressed served as the activation interval (see, e.g., Jauk et al., 2012).

As in other studies concerning divergent thinking (e.g., Rominger, Reitinger, Seyfried, Schneckenleitner, & Fink, 2017), TRP scores were quantified for upper alpha power (10–12 Hz) for an electrode *i* by subtracting the log-transformed power of the reference period (Pow_*i, reference*_) from that of the activation period (Pow_*i, activation*_) for each trial *j* separately, according to the formula:

$$ {\mathrm{TRP}}_i=\mathrm{Median}\left(\log {\left({\mathrm{Pow}}_{i, activation}\right)}_j-\log {\left({\mathrm{Pow}}_{i, reference}\right)}_j\right) $$

Negative values indicate a decrease of task-related alpha power from the reference to the activation period, while positive values express a power increase (Pfurtscheller & Lopes da Silva, 1999).

Changes in task-related functional coupling (i.e., TRPL) were calculated between all intra-hemispheric pairs of frontal and parietal-occipital electrodes (resulting in nine pairs per hemisphere; left: FP1-P3, F3-P3, F7-P3, FP1-P7, F3-P7, F7-P7, FP1-O1, F3-O1, F7-O1; right: FP2-P4, F4-P4, F8-P4, FP2-P8, F4-P8, F8-P8, FP2-O2, F4-O2, F8-O2). After Fisher’s z transformation, TRPL values were calculated by the same formula used to identify the TRP scores (see, Grabner, Fink, & Neubauer, 2007; Neubauer & Fink, 2009; Reiser et al., 2012 for a similar approach). All resulting TRPL values were averaged per hemisphere in order to estimate the mean intra-hemispheric task-related changes of frontal-parietal coupling in *a priori* defined anatomical clusters corresponding to left and right frontal and posterior association cortex regions (see, e.g., Miskovic & Schmidt, 2010; Papousek et al., 2014; Papousek, Ruch et al., 2017; Reiser et al., 2012; Terhune, Cardeña, & Lindgren, 2011 for similar aggregation procedures). The involvement of these cortical sites and the increase in their communication/cooperation is well documented during creative thinking (Fink & Benedek, 2014; Jausovec & Jausovec, 2000; Petsche, 1996).

Volume conduction artifacts should not have been an issue. All distances between two electrodes in the used pairs spanned large distances (Lachaux, et al., 1999; Srinivasan, Winter, Ding, Nunez, 2007). Furthermore, the applied task-related design is well suited to control for potential spurious synchrony in signals. If the activation period contains spurious synchronization, this will be further diminished by subtracting phase-locking values of the reference period from the activation period (both containing a similar amount of spurious synchronization). Positive TRPL values indicate an increase in functional coupling from reference to activation and negative TRPL values represent a decrease in communication between frontal and posterior cortical sites.

In order to calculate the time-course of TRP and TRPL, each activation interval was divided into three isochronous time intervals for each answer of a participant (AU-T1, AU-T2, AU-T3). Only activation periods with at least 250 ms artifact-free EEG-recording per time interval and reference periods with a minimum of 1,000 ms artifact-free data were considered valid and used for statistical analyses. The statistical analyses were only run for those participants (*n* = 86) who produced at least ten activation trials for each time interval that were within the defined data quality range (valid trials for AU-T1: *M* = 14.45, *SD* = 1.40, AU-T2: *M* = 14.19, *SD* = 1.38, AU-T3: *M* = 14.14, *SD* = 1.57, and Reference: *M* = 15.92, *SD* = 0.71). The length of the activation intervals ranged from minimum = 816 ms to maximum = 14,000 ms (*M* = 5859.46 ms, *SD* = 2982.70 ms; reaction time minus 500 ms after stimulus onset and 500 ms before button press).

### Statistical analysis

Analysis of TRP scores was conducted by an 8 × 2 × 3 analysis of variance with the within-subjects factors AREA (eight electrode positions in each hemisphere), HEMISPHERE (left, right), and TIME (AU-T1, AU-T2, AU-T3). The impact of individual differences in task performance on the TRP results was investigated by the same analysis of variance design; however, in addition the continuous between-subjects factor ORIGINALITY of generated ideas was considered as a factor. Functional connectivity values (averaged frontal-parietal TRPL values) were analyzed by a 2 × 3 analysis of variance involving the within-subjects factors HEMISPHERE and TIME. The impact of individual differences in task performance on the TRPL results was also investigated by a separate analysis of variance, additionally considering the continuous between-subjects factor ORIGINALITY of generated ideas. For illustration of interaction effects between TIME and the continuous between-subjects variable, predicted TRP scores and predicted TRPL values were calculated for one standard deviation below and one standard deviation above the sample mean of task performance (i.e., originality of ideas) using standard regression analysis. For illustration purposes only, the percentage change score for the time-interval *j* was calculated according to the formula $\frac{A_{(j)}-R}{R}\ast 100$, separately for power and phase-locking values of each participant. *A*_(*j*)_ indicates power/phase locking values at time-interval *j* and *R* indicates power/phase locking values during the reference period. The predicted percentage change scores were calculated for one standard deviation below and one standard deviation above the sample mean of task performance (i.e., originality of ideas) using standard regression analysis.

Additional Pearson correlations (*r*) were conducted to indicate that originality was independent of reaction time and activation trial length. For EEG sensitivity analyses only, analyses of variance were run for reference and activation, separately. For these analyses power values (log transformed) and PLVs (Fisher’s z transformed) were used as dependent variables. The two analyses of the activation period (with power values and PLVs) – in contrast to the two analyses of the refence period – were run with the additional within-subjects factor TIME. For all sensitivity analyses the originality score served as the between-subjects variable.

In case of violations of sphericity assumptions, a multivariate approach was used (see Vasey & Thayer, 1987). Estimates of effect size are reported using partial eta-squared (*η*_*p*_^*2*^). *Post hoc* comparisons were performed using Tukey’s Honestly Significant Differences (HSD) tests. A significance level of *p* < .05 (two-tailed) was used for all analyses.

## Results

### EEG results

#### Time-course of alpha power during creative ideation

**Fig. 2:**
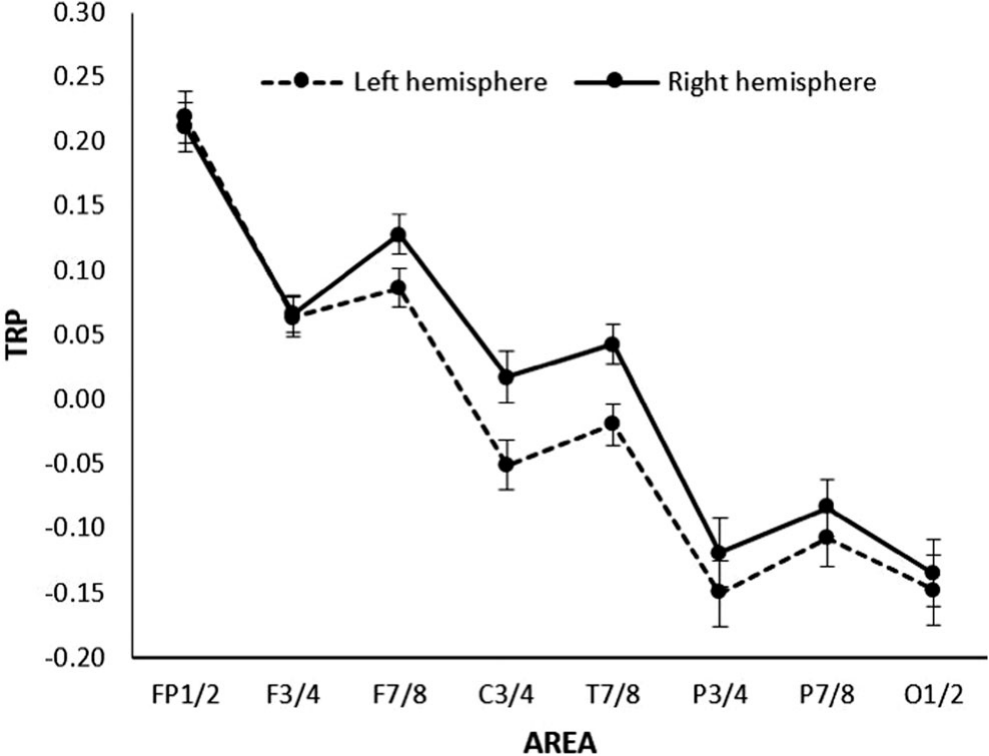
General pattern of task-related power changes (TRP; along with error bars indicating SEM) in the upper alpha band during verbal creative ideation

The 8 × 2 × 3 analysis of variance revealed a significant main effect AREA (*F*(7,79) = 39.86, *p* < .001, *η*_*p*_^*2*^ = .61), indicating decreased alpha power during creative ideation especially over posterior and occipital cortical sites (P3/4, P7/8, O1/2) and increased alpha power at frontal sites (Fp1/2, F3/4, F7/8; see Fig. 2). The main effect HEMISPHERE (*F*(1,85) = 17.34, *p* < .001, *η*_*p*_^*2*^ = .17) indicated relatively higher TRP in the right (*M* = 0.016, *SE* = 0.017) compared to the left hemisphere (*M* = -0.013, *SE* = 0.017). The interaction AREA × HEMISPHERE was also significant (*F*(7,79) = 3.99, *p =* .001, *η*_*p*_^*2*^ = .08), suggesting that the hemispheric difference was apparent at the lateral frontal, central, and temporal positions (F7/8, C3/4, T7/8).

As revealed by the significant main effect TIME (*F*(2,84) = 3.88, *p* = .024, *η*_*p*_^*2*^ = .05), the highest alpha power increase was observed at the final time interval of creative ideation, while the first two intervals were not significantly different (AU-T1: *M* = -0.009, *SE* = 0.017; AU-T2: *M* = -0.007, *SE* = 0.018; AU-T3: *M* = 0.021, *SE* = 0.018). The time-course of alpha power was most pronounced at frontal (Fp1/2, F3/4) and occipital sites (O1/2), as indicated by a significant interaction of AREA × TIME (*F*(14,72) = 3.10, *p* = .001, *η*_*p*_^*2*^ = .06).

**Fig. 3:**
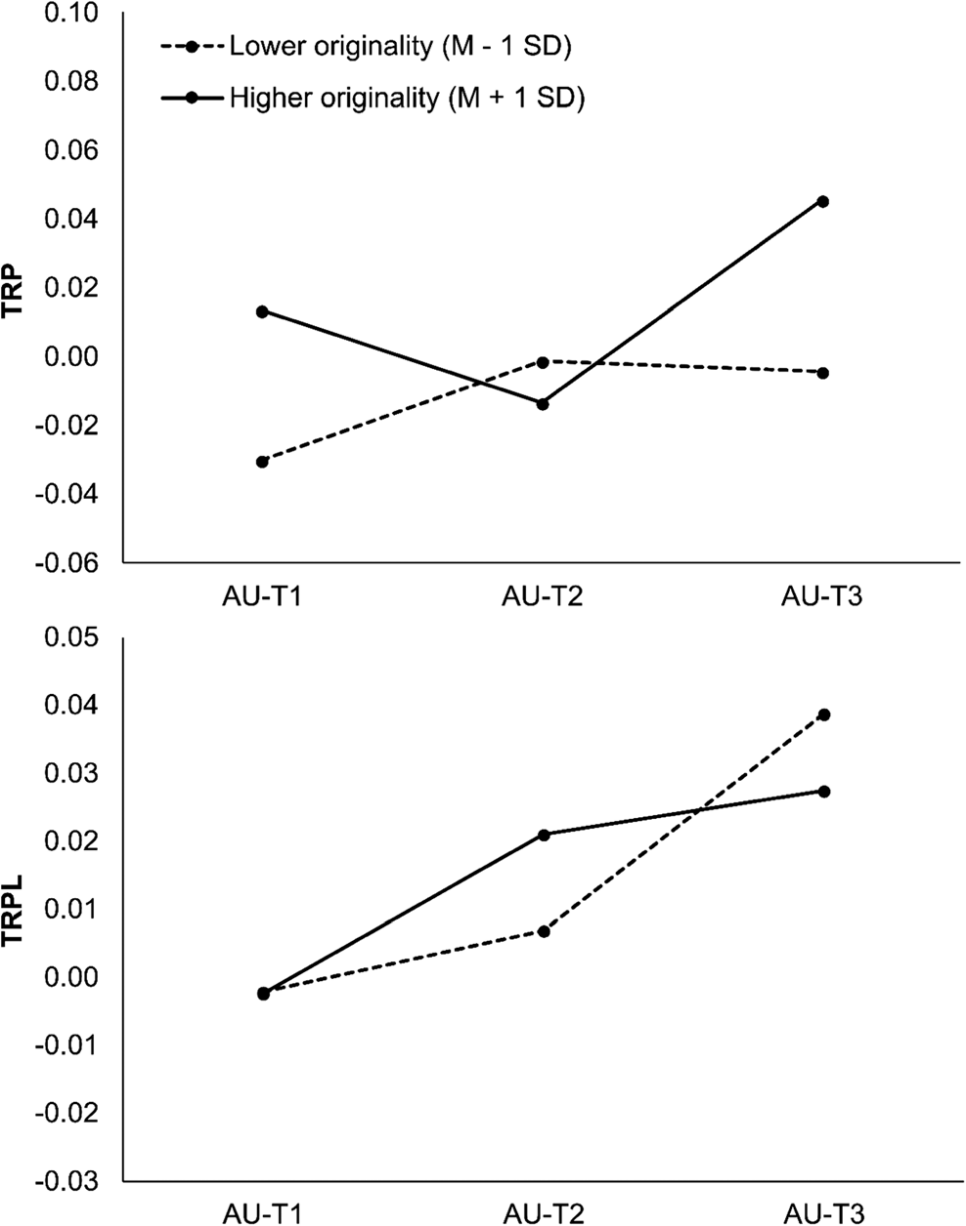
Task-related power (TRP) and task-related phase-locking (TRPL) in the upper alpha band as a function of time and originality

The analysis of variance with ORIGINALITY as continuous between-subjects factor showed a significant interaction between ORIGINALITY and TIME (*F*(2,83) = 8.53, *p <* .001, *η*_*p*_^*2*^ = .06). As illustrated in Fig. 3, more original people showed increased alpha power during the first and the final time interval (+ 17% in AU-T1 and + 28% in AU-T3) of the creative ideation process, whereas less original people showed no U-shape of TRP changes across time. No other effect involving the factor ORIGINALITY was significant.^1^

#### Time-course of phase-locking values (functional coupling) in the alpha band during creative ideation

The 2 × 3 analysis of variance revealed a significant main effect TIME (*F*(2,84) = 14.81, *p* < .001, *η*_*p*_^*2*^ = .21), indicating a continuous increase in functional coupling from AU-T1 (*M* = -0.002, *SE* = 0.004) over AU-T2 (*M* = 0.014, *SE* = 0.006) to AU-T3 (*M* = 0.033, *SE* = 0.007). The main effect HEMISPHERE was also significant (*F*(1,85) = 4.43, *p* = .038, *η*_*p*_^*2*^ = .05), indicating higher TRPL in the left (*M* = 0.019, *SE* = 0.005) compared to the right (*M* = 0.011, *SE* = 0.005) hemisphere. The interaction HEMISPHERE × TIME was not significant (*F*(1,84) = 1.61, *p* = .21, *η*_*p*_^*2*^ = .02).

The analysis of variance involving the continuous between-subjects variable ORIGINALITY indicated a significant interaction between ORIGINALITY and TIME (*F*(2,83) = 4.74, *p =* .011, *η*_*p*_^*2*^ = .03). As illustrated in Fig. 3, more and less original people did not differ in TRPL at the beginning (AU-T1) and the end (AU-T3) of the creative ideation process. Critically, higher originality was associated with a more rapid increase in TRPL from the first (AU-T1 with +0%) to the second time interval (+ 12% in AU-T2), while participants who generated lower original ideas showed relatively stronger increases at later stages (i.e., from AU-T2 with 4% increases to AU-T3 with 19% increases). No other effect involving the factor ORIGINALITY was significant (ORIGINALITY: *F*(1,84) = 0.008, *p* = .928, *η*_*p*_^*2*^ = .00).^1^

### Additional analyses

The originality score was neither associated with the time until the IDEA button was pressed (*r* = .06, *p* = .61) nor with the available sample points per time interval (AU-T1: *r* = -.02, *p* = .83, AU-T2: *r* = .04, *p* = .74, AU-T3: *r* = .04, *p* = .71).

Sensitivity analyses of pre-stimulus reference power revealed only one significant interaction effect involving ORIGINALITY (ORIGINALITY × HEMISPHERE (*F*(1,84) = 4.74, *p* = .032, *η*_*p*_^*2*^ = .05). Importantly, analyses of power values of the activation period showed very similar findings to those found in the TRP analyses – i.e., significant interaction ORIGINALITY × TIME (*F*(2,83) = 10.07, *p* < .001, *η*_*p*_^*2*^ = .06). The sensitivity analyses with PLVs of pre-stimulus reference period as dependent variable revealed a significant main effect ORIGINALITY (*F*(1,84) = 6.56, *p* = .012, *η*_*p*_^*2*^ = .07; no other effect was significant). More original participants showed higher PLVs during the reference period. The analysis for the activation period likewise revealed the interaction ORIGINALITY × TIME, which was found for TRPL (*F*(2,83) = 6.25, *p* = .003, *η*_*p*_^*2*^ = .04; ORIGINALITY: *F*(1,84) = 6.76, *p* = .011, *η*_*p*_^*2*^ = .07).

## Discussion

As hypothesized, the process of creative ideation followed a characteristic time-course of task-related alpha power changes. Specifically, participants who generated more original ideas showed a U-shaped time-course of TRP, with higher alpha power at the beginning and towards the end of the creative ideation process (cf. Schwab et al., 2014). These alpha power changes were accompanied by a steady increase in task-related long distance functional coupling between frontal and parietal-occipital sites, and increases were more rapid in more creative versus less creative people.

Beyond providing a replication of existing findings of more frontal and right (posterior) alpha power during creative ideation (Fink & Benedek, 2014) as well as a time-course of alpha power (Schwab et al., 2014), the central novelty of the present study is the combined use of a functional brain connectivity measure (i.e., phase-locking; Lachaux et al., 1999) and task-related alpha power change scores (Fink & Benedek, 2014; Pfurtscheller & Lopes da Silva, 1999). The approach of combining two largely independent indices of power and phase (i.e., TRP and TRPL; Lachaux et al., 1999; Varela et al., 2001), allows the time-course of neurocognitive processes involved in creative ideation to be studied in more detail. During the first stage of creative ideation, the initial increase in alpha power may reflect an early stage of undirected, associative thinking, where attention is shifted from stimulus processing to internal processes such as the retrieval of associative information from memory (Benedek, Jauk, et al., 2014; Benedek & Jauk, 2018; Gilhooly et al., 2007; Schwab et al., 2014; Silvia et al., 2017). This interpretation is strengthened by the finding of a relatively small increase in task-related phase-locking during this stage. The first stage of the creative thinking process might hence be characterized by relatively low executive demands because of reduced cooperation between frontal and parietal areas and low working memory load similar to resting state conditions. While functional coupling at the first stage of creative thinking was largely independent from originality performance, alpha power was higher in more than in less original people. The latter suggests that a stronger internal focus of attention already at this early stage of the creative process may facilitate more effective memory search and semantic retrieval processes essential to high creative performance (Benedek, Jauk, et al., 2014; 2018; Gilhooly et al., 2007; Schwab et al., 2014).

The second stage seems to involve a change in processes as evidenced by diminished alpha activity and increases in task-related frontal-parietal phase-locking. At this stage semantic content from memory might have already been retrieved and is now integrated to simulate and create possible response alternatives (Gilhooly et al., 2007; Silvia et al., 2017). The decreased alpha power was accompanied by a rise in functional coupling and neuronal integration of long-distant areas, which may reflect the onset of central executive control processes (Beaty & Silvia, 2012; Gilhooly et al., 2007). Strikingly, more original people seemed to show an earlier increase in cooperation between frontal and parietal sites, which suggests earlier recruitment of the executive network, possibly providing control mechanisms relevant for more effective and demanding task strategies (Beaty et al., 2016; Nusbaum & Silvia, 2011; Sauseng et al., 2005; Terhune et al., 2011). The more rapid recruitment of control functions may expand available executive resources in order to evaluate and elaborate the generated ideas, which might further increase their originality (Rominger, Papousek, Perchtold, et al., 2018).

This phase of transition is finally followed by a stage exhibiting the highest level of functional coupling in combination with a marked re-increase in alpha power, which indicates a high prevalence of executive control functions operating under increased internal attention demands. The observed combination of increased power and functional coupling suggests that evaluative and elaboration functions are fully pronounced at this final stage (Jaarsveld et al., 2015; Schwab et al., 2014). Specifically, according to the findings of this study, participants who generated more original ideas may have shown more effective shielding of irrelevant information and increased internal attention supporting complex, vivid mental simulations necessary to evaluate the feasibility and appeal of ideas (Ellamil et al., 2012; Jaarsveld et al., 2015; Rominger, Papousek, Perchtold, et al., 2018). In contrast, although less original people showed a similar level of functional coupling at this final stage of idea generation (i.e., strong involvement of the central executive network), they may be less capable of focusing attentional resources on relevant internal processes. This admittedly tentative interpretation is in accordance with literature reporting increased functional coupling in the executive network at later stages of creative thinking as an index of increased executive control during idea evaluation and elaboration (Beaty et al., 2015, 2016, 2018).

Some limitations of this study need to be addressed. The specificity of the results would be further strengthened if the study design also involved control tasks outside the realm of creativity. However, the task-related approach utilized in this study has the advantage that the neurophysiological activation during a period of creative thinking (i.e., cognitive load) is contrasted with the activation pattern during a period of reference (i.e., no/less cognitive load), which controls for task-unspecific interindividual differences in brain activity. Furthermore, the creative thinking performance during the task (i.e., originality) was associated with a marked u-shaped function and an earlier increase in long distance phase synchronization in the upper alpha band. These associations between neurophysiological changes and task performance strongly indicate the specificity of the reported findings, which was additionally strengthened by the fact that this neurophysiological pattern was specifically found in the upper alpha band and not in the adjacent lower alpha and beta bands. Additional sensitivity analyses indicated that more original participants already showed higher long distance phase synchronization during the reference (and activation) period. Although this finding needs to be replicated in future studies, it may nicely correspond with the study by Beaty et al. (2014), who found increased involvement of executive brain networks at rest in more creative participants. However, it is important to note that the identified pattern of finding in the reference period cannot be responsible for the specific time-course of PLVs and band power, which is the focus of the present study. Like all EEG studies, this study might be also concerned with the exact source localization of activity. In this particular context it would be highly desirable to see some conjoint EEG and fMRI studies in future that apply the same experimental task to the same sample of participants. In this way we would be able to better integrate the spatial and dynamic properties associated with the generation of creative ideas.

However, by combining two different measures of brain functioning (i.e., power and phase-locking), this study corroborates the notion that creative idea generation involves discriminable stages (e.g., retrieval, integration/simulation, evaluation; Benedek, 2018b; see also Ellamil et al., 2012; Fink, Rominger et al., 2018; Finke et al., 1996; Jaarsveld et al., 2015; Rominger, Papousek, Perchtold, et al., 2018; Schwab et al., 2014), and high creative potential is associated with characteristic differences at each stage. At the level of alpha activity, more creative people showed a U-shaped time course with increased alpha activity at the beginning and the end of idea generation that has been previously related to the production of more original ideas (Schwab et al., 2014). At the level of frontal-parietal phase-locking in the upper alpha range, more creative people showed a more rapid increase, suggesting an earlier involvement of relevant executive control mechanisms (Benedek, Jauk, et al., 2014; Nusbaum & Silvia, 2011).

Taken together, the results of this study add new evidence to the notion that the temporal dynamics of neuro-cognitive functions across the creative thinking process affect the creativity of the outcome.

## Footnotes

1 In order to examine the specificity of the reported effects for the upper alpha band, the same analyses were re-run in the lower alpha band (8–10 Hz) and the beta band (12–30 Hz). Importantly, the time-by-orignality interaction did not reach significance in these frequency bands. With the exception of a weak but significant four-way interaction in the ANOVA with the TRP scores in the lower alpha band as dependent variable (TIME × POSITION × HEMISPHERE × ORIGINALITY) with a rather low effect size (*η*_*p*_^*2*^=.01), there were no significant effects involving originality.

## References

[CR1] Amabile TM (1982). Social psychology of creativity: A consensual assessment technique. Journal of Personality and Social Psychology.

[CR2] Arden R, Chavez RS, Grazioplene R, Jung RE (2010). Neuroimaging creativity: A psychometric view. Behavioural Brain Research.

[CR3] Beaty RE (2015). The neuroscience of musical improvisation. Neuroscience and Biobehavioral Reviews.

[CR4] Beaty RE, Benedek M, Kaufman BS, Silvia PJ (2015). Default and executive network coupling supports creative idea production. Scientific Reports.

[CR5] Beaty RE, Benedek M, Silvia PJ, Schacter DL (2016). Creative cognition and brain network dynamics. Trends in Cognitive Sciences.

[CR6] Beaty, R. E., Benedek, M., Wilkins, R. W., Jauk, E. V., Fink, A., Silvia, P. J., . . . Neubauer, A. C. (2014). Creativity and the default network: A functional connectivity analysis of the creative brain at rest. *Neuropsychologia, 64C,* 92–98. 10.1016/j.neuropsychologia.2014.09.019 PMC4410786 25245940

[CR7] Beaty, R. E., Kenett, Y. N., Christensen, A. P., Rosenberg, M. D., Benedek, M., Chen, Q., . . . Silvia, P. J. (2018). Robust prediction of individual creative ability from brain functional connectivity. *Proceedings of the National Academy of Sciences of the United States of America, 115,* 1087–1092. 10.1073/pnas.1713532115 10.1073/pnas.1713532115 PMC5798342 29339474

[CR8] Beaty RE, Silvia PJ (2012). Why do ideas get more creative across time? An executive interpretation of the serial order effect in divergent thinking tasks. Psychology of Aesthetics, Creativity, and the Arts.

[CR9] Benedek M, Jung RE, Vartanian O (2018). Internally directed attention in creative cognition. *The Cambridge handbook of the neuroscience of creativity*.

[CR10] Benedek, M. (2018b). The neuroscience of creative idea generation. In Z. Kapoula, E. Volle, J. Renoult, & M. Andreatta (Eds.), *Exploring transdisciplinarity in art and sciences*. Springer.

[CR11] Benedek M, Bergner S, Könen T, Fink A, Neubauer AC (2011). EEG alpha synchronization is related to top-down processing in convergent and divergent thinking. Neuropsychologia.

[CR12] Benedek M, Jauk E, Fox KCR, Christoff K (2018). Spontaneous and controlled processes in creative cognition. *The Oxford handbook of spontaneous thought. Mind-wandering, creativity, and dreaming*.

[CR13] Benedek M, Jauk E, Fink A, Koschutnig K, Reishofer G, Ebner F, Neubauer AC (2014). To create or to recall? Neural mechanisms underlying the generation of creative new ideas. NeuroImage.

[CR14] Benedek M, Schickel RJ, Jauk EV, Fink A, Neubauer AC (2014). Alpha power increases in right parietal cortex reflects focused internal attention. Neuropsychologia.

[CR15] Benedek M, Schües T, Beaty RE, Jauk E, Koschutnig K, Fink A, Neubauer AC (2018). To create or to recall original ideas: Brain processes associated with the imagination of novel object uses. Cortex.

[CR16] Boccia M, Piccardi L, Palermo L, Nori R, Palmiero M (2015). Where do bright ideas occur in our brain? Meta-analytic evidence from neuroimaging studies of domain-specific creativity. Frontiers in Psychology.

[CR17] Cheng L, Hu W, Jia X, Runco MA (2016). The different role of cognitive inhibition in early versus late creative problem finding. Psychology of Aesthetics, Creativity, and the Arts.

[CR18] Ellamil M, Dobson C, Beeman M, Christoff K (2012). Evaluative and generative modes of thought during the creative process. NeuroImage.

[CR19] Fink, A., Bay, J., Koschutnig, K., Rominger, C., Benedek, M., Papousek, I., . . . Memmert, D. (2018). Brain and soccer: Functional patterns of brain activity during the generation of creative moves in real soccer decision-making situations. *Submitted for publication*, 10.1002/hbm.24408 PMC6492000 30259600

[CR20] Fink A, Benedek M (2014). EEG alpha power and creative ideation. Neuroscience and Biobehavioral Reviews.

[CR21] Fink A, Benedek M, Grabner RH, Staudt B, Neubauer AC (2007). Creativity meets neuroscience: Experimental tasks for the neuroscientific study of creative thinking. Methods.

[CR22] Fink A, Graif B, Neubauer AC (2009). Brain correlates underlying creative thinking: EEG alpha activity in professional vs. novice dancers. NeuroImage.

[CR23] Fink A, Perchtold C, Rominger C, Jung RE, Vartanian O (2018). Creativity and cognitive control in the cognitive and affective domains. *The Cambridge handbook of the neuroscience of creativity*.

[CR24] Fink, A., Rominger, C., Benedek, M., Perchtold, C. M., Papousek, I., Weiss, E. M., . . . Memmert, D. (2018). EEG alpha activity during imagining creative moves in soccer decision-making situations. *Neuropsychologia,* 118–124. 10.1016/j.neuropsychologia.2018.04.025 10.1016/j.neuropsychologia.2018.04.025 29702162

[CR25] Fink, A., Weiss, E. M., Schwarzl, U., Weber, H., Assunção, V. L. de, Rominger, C., . . . Papousek, I. (2017). Creative ways to well-being: Reappraisal inventiveness in the context of anger-evoking situations. *Cognitive, Affective & Behavioral Neuroscience, 17,* 94–105. 10.3758/s13415-016-0465-9 10.3758/s13415-016-0465-9 PMC5272882 27683302

[CR26] Finke RA, Ward TB, Smith SM (1996). *Creative cognition: Theory, research, and applications*.

[CR27] Gilhooly KJ, Fioratou E, Anthony SH, Wynn V (2007). Divergent thinking: Strategies and executive involvement in generating novel uses for familiar objects. British Journal of Psychology.

[CR28] Goldschmidt G (2016). Linkographic evidence for concurrent divergent and convergent thinking in creative design. Creativity Research Journal.

[CR29] Gonen-Yaacovi G, de Souza LC, Levy R, Urbanski M, Josse G, Volle E (2013). Rostral and caudal prefrontal contribution to creativity: A meta-analysis of functional imaging data. Frontiers in Human Neuroscience.

[CR30] Grabner RH, Fink A, Neubauer AC (2007). Brain correlates of self-rated originality of ideas: Evidence from event-related power and phase-locking changes in the EEG. Behavioral Neuroscience.

[CR31] Guilford JP (1967). *The nature of human intelligence*.

[CR32] Jaarsveld S, Fink A, Rinner M, Schwab D, Benedek M, Lachmann T (2015). Intelligence in creative processes: An EEG study. Intelligence.

[CR33] Jauk EV, Benedek M, Neubauer AC (2012). Tackling creativity at its roots: Evidence for different patterns of EEG alpha activity related to convergent and divergent modes of task processing. International Journal of Psychophysiology.

[CR34] Jausovec N (2000). Differences in cognitive processes between gifted, intelligent, creative, and average individuals while solving complex problems: An EEG study. Intelligence.

[CR35] Jausovec N, Jausovec K (2000). EEG activity during the performance of complex mental problems. International Journal of Psychophysiology.

[CR36] Jung RE, Vartanian O (2018). *The Cambridge handbook of the neuroscience of creativity*.

[CR37] Kleinmintz, O. M., Abecasis, D., Tauber, A., Geva, A., Chistyakov, A. V., Kreinin, I., . . . Shamay-Tsoory, S. G. (2018). Participation of the left inferior frontal gyrus in human originality. *Brain Structure & Function, 223,* 329–341*.*10.1007/s00429-017-1500-5 10.1007/s00429-017-1500-5 28828749

[CR38] Lachaux J-P, Rodriguez E, Martinerie J, Varela FJ (1999). Measuring phase synchrony in brain signals. Human Brain Mapping.

[CR39] Lopata JA, Nowicki EA, Joanisse MF (2017). Creativity as a distinct trainable mental state: An EEG study of musical improvisation. Neuropsychologia.

[CR40] Miskovic, V., & Schmidt, L. A. (2010). Cross-regional cortical synchronization during affective image viewing. *Brain Research,* 102–111. 10.1016/j.brainres.2010.09.102 10.1016/j.brainres.2010.09.102 20920492

[CR41] Neubauer AC, Fink A (2009). Intelligence and neural efficiency: Measures of brain activation versus measures of functional connectivity in the brain: Intelligence and the Brain. Intelligence.

[CR42] Nusbaum EC, Silvia PJ (2011). Are intelligence and creativity really so different? Fluid intelligence, executive processes, and strategy use in divergent thinking. Intelligence.

[CR43] Papousek, I., Ruch, W., Rominger, C., Kindermann, E., Scheidl, K., Schulter, G., . . . Weiss, E. M. (2017). The use of bright and dark types of humour is rooted in the brain. *Scientific Reports, 7,* 42967. 10.1038/srep42967 10.1038/srep42967 PMC5314334 28211496

[CR44] Papousek I, Weiss EM, Mosbacher JA, Reiser EM, Schulter G, Fink A (2014). Affective processing in positive schizotypy: Loose control of social-emotional information. Brain and Cognition.

[CR45] Papousek, I., Weiss, E. M., Perchtold, C. M., Weber, H., Assuncao, V. L. de, Schulter, G., . . . Fink, A. (2017). The capacity for generating cognitive reappraisals is reflected in asymmetric activation of frontal brain regions. *Brain Imaging and Behavior, 11,* 577–590. 10.1007/s11682-016-9537-2 10.1007/s11682-016-9537-2 PMC5408052 26935554

[CR46] Perchtold CM, Papousek I, Koschutnig K, Rominger C, Weber H, Weiss EM, Fink A (2018). Affective creativity meets classic creativity in the scanner. Human Brain Mapping.

[CR47] Petsche H (1996). Approaches to verbal, visual and musical creativity by EEG coherence analysis. International Journal of Psychophysiology.

[CR48] Petsche H, Kaplan S, von Stein A, Filz O (1997). The possible meaning of the upper and lower alpha frequency ranges for cognitive and creative tasks. International Journal of Psychophysiology.

[CR49] Pfurtscheller G, Lopes da Silva FH (1999). Event-related EEG/MEG synchronization and desynchronization: Basic principles. Clinical Neurophysiology.

[CR50] Pidgeon, L. M., Grealy, M., Duffy, A. H. B., Hay, L., McTeague, C., Vuletic, T., . . . Gilbert, S. J. (2016). Functional neuroimaging of visual creativity: A systematic review and meta-analysis. *Brain and Behavior* e00540. 10.1002/brb3.540 10.1002/brb3.540 PMC5064346 27781148

[CR51] Pinho AL, Ullen F, Castelo-Branco M, Fransson P, de Manzano O (2016). Addressing a paradox: Dual strategies for creative performance in introspective and extrospective networks. Cerebral Cortex.

[CR52] Pringle A, Sowden PT (2017). Unearthing the creative thinking process: Fresh insights from a think-aloud study of garden design. Psychology of Aesthetics, Creativity, and the Arts.

[CR53] Reiser EM, Schulter G, Weiss EM, Fink A, Rominger C, Papousek I, Reiser EM (2012). Decrease of prefrontal–posterior EEG coherence: Loose control during social–emotional stimulation. Brain and Cognition.

[CR54] Rominger C, Fink A, Weiss EM, Bosch J, Papousek I (2017). Allusive thinking (remote associations) and auditory top-down inhibition skills differentially predict creativity and positive schizotypy. Cognitive Neuropsychiatry.

[CR55] Rominger C, Papousek I, Perchtold CM, Weber B, Weiss EM, Fink A (2018). The creative brain in the figural domain: Distinct patterns of EEG alpha power during idea generation and idea elaboration. Neuropsychologia.

[CR56] Rominger C, Papousek I, Weiss EM, Schulter G, Perchtold CM, Lackner HK, Fink A (2018). Creative thinking in an emotional context: Specific relevance of executive control of emotion-laden representations in the inventiveness in generating alternative appraisals of negative events. Creativity Research Journal.

[CR57] Rominger C, Reitinger J, Seyfried C, Schneckenleitner E, Fink A (2017). The reflecting brain: Reflection competence in an educational setting is associated with increased electroencephalogram activity in the alpha band. Mind, Brain, and Education.

[CR58] Runco MA, Acar S (2012). Divergent thinking as an indicator of creative potential. Creativity Research Journal.

[CR59] Sauseng P, Klimesch W, Doppelmayr M, Pecherstorfer T, Freunberger R, Hanslmayr S (2005). EEG alpha synchronization and functional coupling during top-down processing in a working memory task. Human Brain Mapping.

[CR60] Schwab D, Benedek M, Papousek I, Weiss EM, Fink A (2014). The time-course of EEG alpha power changes in creative ideation. Frontiers in Human Neuroscience.

[CR61] Silvia PJ, Beaty RE, Nusbaum EC, Eddington KM, Kwapil TR (2014). Creative motivation: Creative achievement predicts cardiac autonomic markers of effort during divergent thinking. Biological Psychology.

[CR62] Silvia PJ, Nusbaum EC, Beaty RE (2017). Old or new? Evaluating the old/new scoring method for divergent thinking tasks. The Journal of Creative Behavior.

[CR63] Sowden PT, Pringle A, Gabora L (2015). The shifting sands of creative thinking: Connections to dual-process theory. Thinking and Reasoning.

[CR64] Srinivasan R, Winter WR, Ding J, Nunez PL (2007). EEG and MEG coherence: Measures of functional connectivity at distinct spatial scales of neocortical dynamics. Journal of Neuroscience Methods.

[CR65] Steingrüber H-J (2010). *Hand-Dominanz-Test: H-D-T*.

[CR66] Terhune DB, Cardeña E, Lindgren M (2011). Differential frontal-parietal phase synchrony during hypnosis as a function of hypnotic suggestibility. Psychophysiology.

[CR67] Varela F, Lachaux JP, Rodriguez E, Martinerie J (2001). The brainweb: Phase synchronization and large-scale integration. Nature Reviews Neuroscience.

[CR68] Vasey MW, Thayer JF (1987). The continuing problem of false positives in repeated measures ANOVA in psychophysiology: A multivariate solution. Psychophysiology.

[CR69] Wang M, Hao N, Ku Y, Grabner RH, Fink A (2017). Neural correlates of serial order effect in verbal divergent thinking. Neuropsychologia.

[CR70] Zabelina DL, Robinson MD, Council JR, Bresin K (2012). Patterning and nonpatterning in creative cognition: Insights from performance in a random number generation task. Psychology of Aesthetics, Creativity, and the Arts.

